# Inhaled CO_2_ vs. Hypercapnia Obtained by Low Tidal Volume or Instrumental Dead Space in Unilateral Pulmonary Artery Ligation: Any Difference for Lung Protection?

**DOI:** 10.3389/fmed.2022.901809

**Published:** 2022-05-20

**Authors:** Elena Spinelli, Antonio Pesenti, Gianluca Lopez, Anna Damia, Francesco Damarco, Erica Garbelli, Gaia Dal Santo, Alessio Caccioppola, Giorgio Giudici, Virginia Figgiaconi, Osvaldo Biancolilli, Michele Battistin, Caterina Lonati, Valentina Vaira, Lorenzo Rosso, Stefano Ferrero, Stefano Gatti, Tommaso Mauri

**Affiliations:** ^1^Department of Anesthesia, Critical Care and Emergency, Fondazione Istituto di Ricovero e Cura a Carattere Scientifico (IRCCS) Ca' Granda Ospedale Maggiore Policlinico, Milan, Italy; ^2^Department of Pathophysiology and Transplantation, University of Milan, Milan, Italy; ^3^Department of Biomedical Surgical and Dental Sciences, University of Milan, Milan, Italy; ^4^Division of Thoracic Surgery and Lung Transplantation, Fondazione IRCCS Ca' Granda Ospedale Maggiore Policlinico, Milan, Italy; ^5^Center for Preclinical Research, Fondazione IRCCS Ca' Granda, Ospedale Maggiore Policlinico, Milan, Italy; ^6^Division of Pathology, Fondazione IRCCS Ca' Granda Ospedale Maggiore Policlinico, Milan, Italy

**Keywords:** ventilator-induced lung injury, pulmonary perfusion, inhaled CO_2_, therapeutic hypercapnia, mechanical ventilation

## Abstract

**Background:**

Unilateral ligation of the pulmonary artery (UPAL) induces bilateral lung injury in pigs undergoing controlled mechanical ventilation. Possible mechanisms include redistribution of ventilation toward the non-ligated lung and hypoperfusion of the ligated lung. The addition of 5% CO_2_ to the inspiratory gas (FiCO_2_) prevents the injury, but it is not clear whether lung protection is a direct effect of CO_2_ inhalation or it is mediated by plasmatic hypercapnia. This study aims to compare the effects and mechanisms of FiCO_2_ *vs*. hypercapnia induced by low tidal volume ventilation or instrumental dead space.

**Methods:**

Healthy pigs underwent left UPAL and were allocated for 48 h to the following: Volume-controlled ventilation (VCV) with V_T_ 10 ml/kg (injury, *n* = 6); VCV plus 5% FiCO_2_ (FiCO_2_, *n* = 7); VCV with V_T_ 6 ml/kg (low V_T_, *n* = 6); VCV plus additional circuit dead space (instrumental V_D_, *n* = 6). Histological score, regional compliance, wet-to-dry ratio, and inflammatory infiltrate were assessed to evaluate lung injury at the end of the study. To investigate the mechanisms of protection, we quantified the redistribution of ventilation to the non-ligated lung, as the ratio between the percentage of tidal volume to the right and to the left lung (V_TRIGHT/LEFT_), and the hypoperfusion of the ligated lung as the percentage of blood flow reaching the left lung (Perfusion_LEFT_).

**Results:**

In the left ligated lung, injury was prevented only in the FiCO_2_ group, as indicated by lower histological score, higher regional compliance, lower wet-to-dry ratio and lower density of inflammatory cells compared to other groups. For the right lung, the histological score was lower both in the FiCO_2_ and in the low V_T_ groups, but the other measures of injury showed lower intensity only in the FiCO_2_ group. V_TRIGHT/LEFT_ was lower and Perfusion_LEFT_ was higher in the FiCO_2_ group compared to other groups.

**Conclusion:**

In a model of UPAL, inhaled CO_2_ but not hypercapnia grants bilateral lung protection. Mechanisms of protection include reduced overdistension of the non-ligated and increased perfusion of the ligated lung.

## Introduction

Pathologic changes in lung perfusion in acute respiratory failure include a spectrum of functional and anatomical alterations ranging from impaired regional vaso-regulation to perfusion micro-thrombotic defects to pulmonary embolism (1, 2). These changes might contribute to ventilation-induced lung injury (VILI) through several mechanisms, including alveolar hypocapnia (3, 4), inhomogeneous distribution of ventilation (5–7), and regional hypoperfusion (8, 9). Notably, these mechanisms may be at play even when ventilation is delivered within protective limits. In the current clinical practice, prevention of VILI is based on the minimization of the injurious effects of tidal volume and pressure (10), the cornerstone of protective ventilation (11). On the contrary, prevention of VILI through correction of pathological alterations due to ventilation and perfusion inhomogeneity has received little attention.

A previous study conducted by our group showed that the addition of 5% CO_2_ to inspiratory gas prevents bilateral VILI in an experimental model of ligation of the left pulmonary artery (12). Mechanisms of protection included decreased inflammation in both lungs and more homogeneous distribution of ventilation, with reduced overdistension of the right lung.

Inhalation of 5% CO_2_ corrects alveolar hypocapnia in the ligated lung but also induces plasmatic hypercapnia. A few studies showed that plasmatic hypercapnia *per se* exerts anti-inflammatory actions and could prevent lung injury (13–15). Inhaled CO_2_ limits the deleterious consequences of alveolar hypocapnia in the ligated lung dampening pneumoconstriction (16) and surfactant depletion (4, 17). Thus, the question of the mechanism by which CO_2_ protects the lung (through a specific effect of the inhalation route or by plasmatic hypercapnia) still remains unanswered. Moreover, while the addition of CO_2_ to inspiratory gas has the unique potential of correcting alveolar hypocapnia, plasmatic hypercapnia can be obtained by clinically easier methods, such as low tidal volume ventilation or the addition of instrumental dead space.

We designed this experimental study to compare the lung-protective effects of inhalation of 5% CO_2_ *vs*. hypercapnia obtained either by low tidal volume or instrumental dead space in our model of unilateral pulmonary artery ligation. Our hypothesis was that the effects of inhaled CO_2_ might be more comprehensive in the presence of unilateral perfusion block, possibly leading to more effective protection of the lungs. We also explored the mechanisms of protection for each lung by monitoring regional ventilation and perfusion by electrical impedance tomography (EIT).

## Materials and Methods

**Figure 1:**
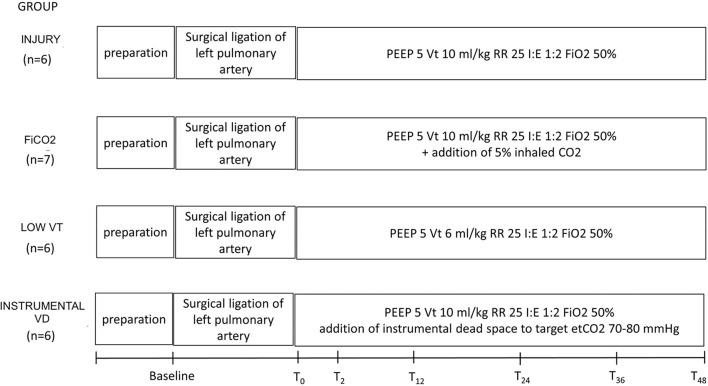
Study design and timeline. Preparation corresponds to anesthesia and invasive monitoring, which took about 1 h. After baseline measurements, animals underwent surgical ligation of the left pulmonary artery. T0 to 48 corresponds to the study period, during which each group received a specific treatment, according to the study group. VT, tidal volume; PEEP, positive end-expiratory pressure in cm H_2_O; RR, respiratory rate in breaths/min.

Figure 1 summarizes the study design. The study was approved by the Italian Ministry of Health (protocol No. 543/2018-PR) and conducted according to the European Directive 2010/63/EU on the protection of animals used for scientific purposes and Italian legislative decree 26/2014. Approval by the Institutional Animal Care Committee was obtained before starting the experiments.

### Animal Preparation

Twenty-five healthy female pigs (36 ± 5 Kg) were anesthetized, intubated through surgical tracheostomy, and ventilated in the prone position using volume-controlled ventilation with tidal volume (V_T_) 10 ml/kg, respiratory rate (RR) 25 bpm, inspiratory/expiratory time ratio (I/E) 1:2, positive end-expiratory pressure (PEEP) 5-cm H_2_O, and FIO_2_ 0.5 (baseline settings).

General anesthesia and neuromuscular blockade were maintained by IV propofol 5–10 mg/kg/h, medetomidine 2.5–10.0 μg/kg/h, and pancuronium bromide 0.3–0.5 mg/kg/h for the whole study period.

After baseline measurements, surgical ligation of the left pulmonary artery was performed as previously described (12). Briefly, a left mini-thoracotomy was performed with the animal in the right lateral position and the main left pulmonary artery was isolated and progressively (5 minutes) occluded and then ligated.

### Study Groups

Right after the ligation procedure, animals were turned prone and allocated to one of four study groups:

Left pulmonary artery ligation (injury, *n* = 6) with the following standard ventilation settings: V_T_ 10 ml/kg, RR 25 bpm, I:E 1:2, PEEP 5 cmH_2_O, FiO_2_ 0.5.

Left pulmonary artery ligation + inhaled CO_2_ (FiCO_2_, *n* = 7) with standard ventilation settings except for inspired gases switched to a mixture of 50% O_2_, 5% CO_2_, and 45% N_2_.

Left pulmonary artery ligation with low tidal volume (low V_T_, *n* = 6) with standard ventilation settings except for V_T_ 6 ml/kg, as recommended by the American Thoracic Society's guidelines of protective ventilation in Acute Respiratory Distress Syndrome (ARDS) patients (18).

Left pulmonary artery ligation with increased instrumental dead space (instrumental V_D_, *n* = 6) with standard ventilation settings plus additional tubing positioned after the circuit Y-targeted to end-tidal CO_2_ of 70–80 mmHg (similar to end-tidal CO_2_ levels obtained in the low V_T_ group).

All animals were ventilated for 48 h.

### Study Measurements

Data from respiratory mechanics, hemodynamics, blood gas analysis, and EIT were collected at baseline and after 2, 12, 24, 36, and 48 h from end of the ligation procedure (T2, T12, T24, T36, T48). The EIT data were recorded and stored for offline analysis by dedicated software (Dräger EIT Data Analysis Tool 6.3, Lübeck, Germany). From EIT ventilation maps we measured regional V_T_ distribution for the right lung (V_TRIGHT_) and left lung (V_TLEFT_), the ratio between the two lungs (V_TRIGHT/LEFT_) and regional respiratory system compliance of each lung as the ratio between regional V_T_ and driving pressure. The EIT perfusion maps were derived from offline analysis of the time–impedance curve obtained during the first pass of a 10-ml bolus of 5% saline solution during end-inspiratory occlusion, as previously described (19), and used to measure the percentage of perfusion to the left lung (Perfusion_LEFT_).

### End of the Experiment

At T48, animals were euthanized, and lung tissue samples were collected for the following:

- Histology: the severity of regional lung injury in each lung was quantified by using a composite histological score, ranging from 0 (no injury) to 30 (severe), as previously described (12).
- Wet-to-dry calculation (12).
- Quantitative immunohistochemical analysis for measuring the percentage of cells positive for myeloperoxidase (MPO, i.e., neutrophils) and allograft inflammatory factor 1 (AIF-1, i.e., macrophages) (20).
- the Terminal deoxynucleotidyl transferase dUTP nick end labeling (TUNEL) assay (ApopTag Plus Peroxidase *In Situ* Apoptosis Kit from Merck-Millipore) was employed to evaluate apoptosis on tissue samples of the left lungs from two representative animals for each study group as previously described (21).

### Sample Size

The difference in histological scoring of the lungs of the four study groups was the primary endpoint of the study. The sample size was similar to the previous animal studies on the same topic (6, 13). However, we performed an exploratory power analysis and we hypothesized, based on our previous study (12), an effect size of 0.75; to obtain the power of 0.8 with alpha 0.05, the minimum sample size resulted in *n* = 6 per group.

### Statistical Analysis

The data are shown as mean ± standard deviation or median [quartiles], as appropriate. The data measured at the end of the experiment were compared using one-way ANOVA or Kruskal–Wallis test, followed by Dunnett or Dunn's test for multiple comparisons. Longitudinal data (physiological and EIT variables along the study time points) was analyzed using repeated measures two-way ANOVA or mixed-effect analysis, as appropriate, with time and group as a covariate. Statistical significance was defined by *p* < 0.05. Analyses were performed using GraphPad Prism 9.

Additional details are available in the online supplement.

## Results

### Alterations of Gas Exchange and Respiratory Mechanics

**Figure 2:**
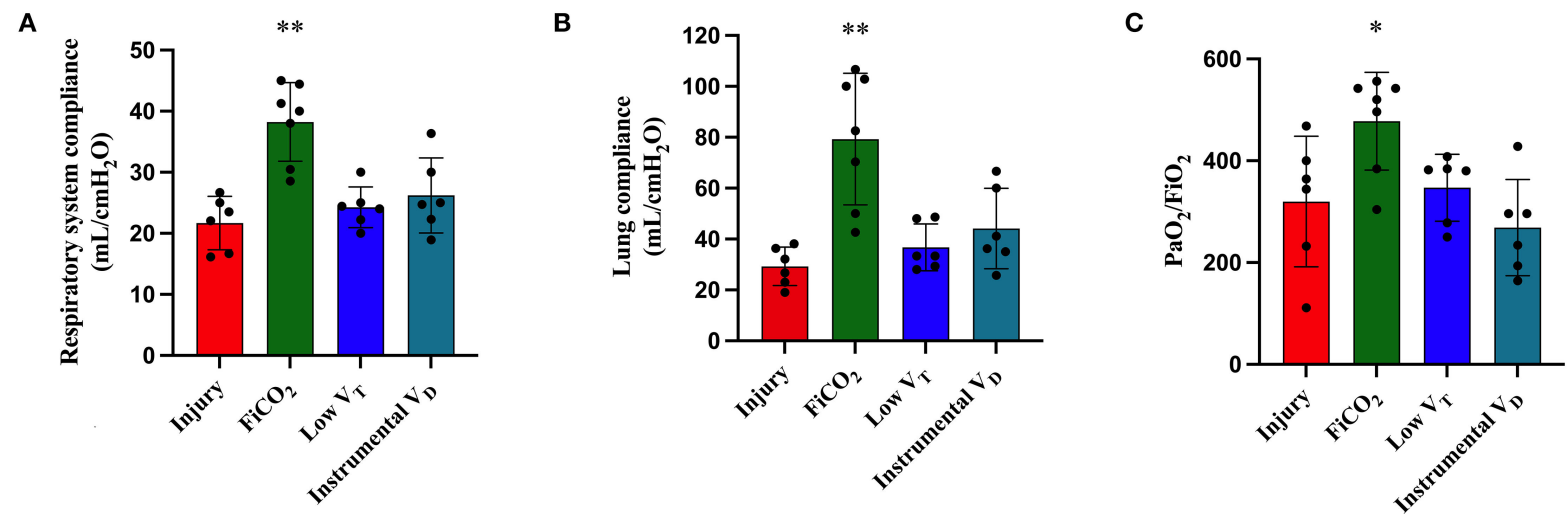
Respiratory mechanics and gas exchanges at the end of the experiment. Respiratory system compliance **(A)**, lung compliance **(B)**, and PaO_2_/FiO_2_ ratio **(C)** were higher in the FiCO_2_ group compared to the other groups. Data are expressed as mean ± SEM. Comparisons are obtained with ordinary one-way ANOVA or Kruskal–Wallis test for normally and non-normally distributed values, respectively, followed by Dunnett or Dunn's multiple comparison test. *p < 0.05, **p < 0.01 vs. Injury group.

At T48, animals in the FiCO_2_ group had significantly higher respiratory system and lung compliance and PaO_2_/FiO_2_ ratio in comparison to all the other study groups. Indeed, the low V_T_ and instrumental V_D_ groups showed global signs of lung injury in terms of decreased compliance of the respiratory system due to decreased lung compliance and decreased PaO_2_/FiO_2_ ratio (Figures 2A–C).

Complete data on respiratory mechanics, blood gases, and hemodynamics at T48 in the four study groups are reported in Supplementary Table S1.

### Protection of the Left Ligated Lung

**Figure 3:**
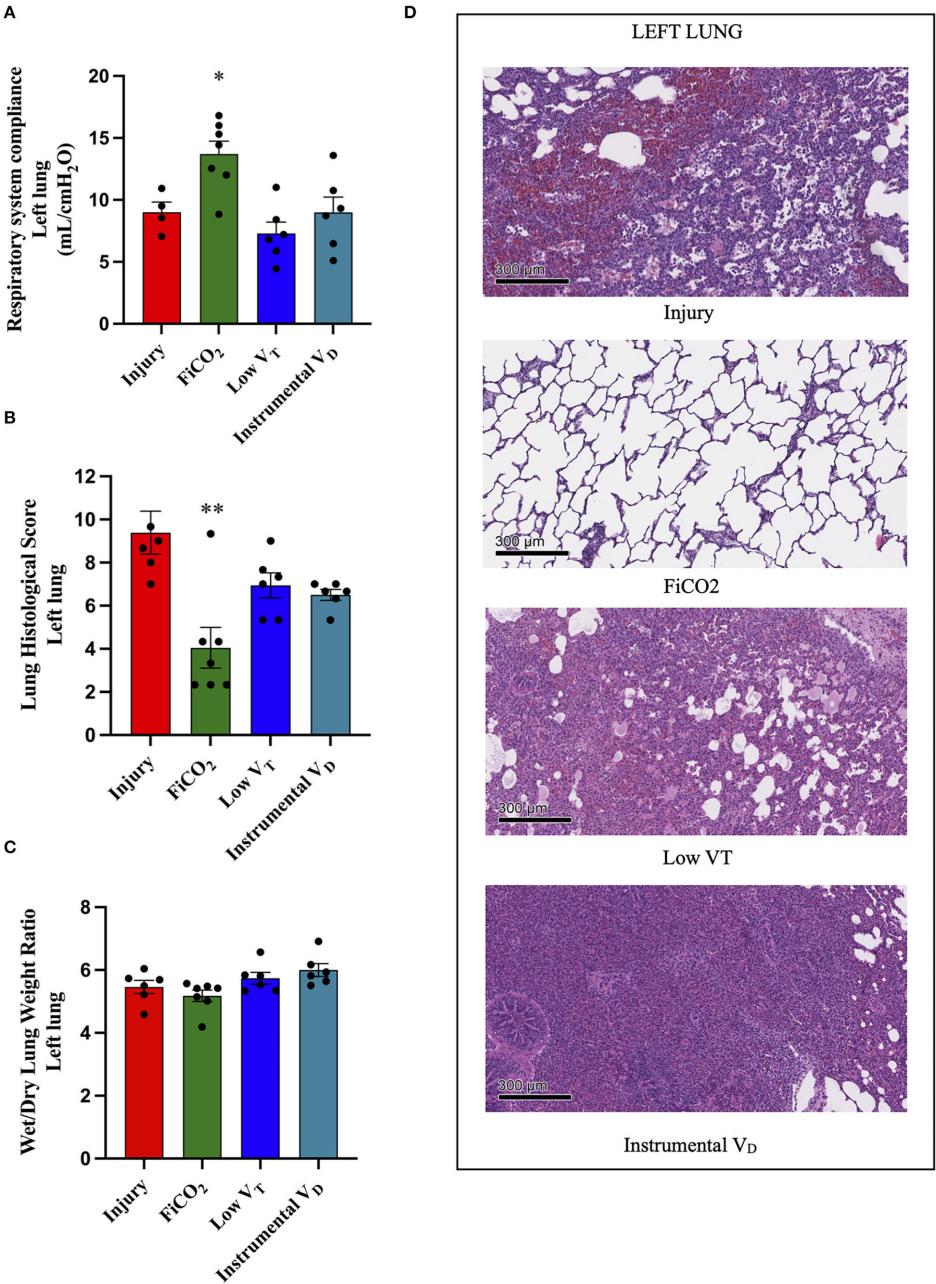
Left lung injury. Left-side respiratory system compliance at the end of the experiment **(A)**. Histological score of left lungs from each study group **(B)**. Wet-to-dry of left lungs **(C)**. Microscopic appearance of the lungs at the end of the experiment **(D)**. Representative microphotographs of the left ligated lungs from the four study groups (H&E, original magnification 100 ×). Data are expressed as scatter dot plots with mean ± SEM. Comparisons are obtained with ordinary one-way ANOVA or Kruskal–Wallis test for normally and non-normally distributed values, respectively, followed by Dunnett or Dunn's multiple comparison test. *p < 0.05, **p < 0.01 vs. Injury group.

The signs of lung injury for the left ligated lung, including left-side respiratory system compliance measured by EIT, histological score, and wet-to dry ratio, were significantly different between the four study groups (*p* = 0.005, *p* = 0.0015, *p* = 0.026, respectively) (Figures 3A–C). The most efficient lung protection was found in the FiCO_2_ group, which showed higher regional compliance [14 (12 – 16) *vs*. 9 (7 – 11), *p* = 0.02] and lower histological score [3 (2 – 4) *vs*. 9 (8 – 11), *p* = 0.01] compared to the injury group.

The left lungs of animals in the FiCO_2_ group showed nearly normal histologic appearance, while injury was evident in all the other study groups. Hemorrhagic areas and inflammatory infiltrate composed mainly of macrophages characterized the injury group; vascular congestion, edema, and inflammatory infiltrate composed of macrophages and lymphocytes were prevalent in the low V_T_ group; extensive consolidation by inflammatory infiltrate composed of macrophages, granulocytes, and lymphocytes described the instrumental V_D_ group (Figure 3D).

Immunohistochemical analyses showed significantly different densities of MPO-positive neutrophils in the left lungs of the four groups (*p* = 0.002). Interestingly, lungs from the FiCO_2_ showed the lowest values, while the low V_T_ and instrumental V_D_ groups had very high values (Table 1 and Supplementary Figure S1).

**Table 1:** Characterization by immunohistochemistry of the lung immune cell infiltrates in the different groups.

	**Injury** **(*n* = 6)**	**FiCO_**2**_** **(*n* = 7)**	**Low V_**T**_** **(*n* = 6)**	**Instrumental V_**D**_** **(*n* = 6)**	***p* value**
**Left lung**
MPO positive cells, %	0.8 [0.5–1.9]	0.4 [0.3–0.5]	11.5 [2.4–33.5]	10.8 [3.4–16.9]	**0.002**
AIF-1 positive cells, %	54 [34–62]	26 [24–38]	45 [32–56]	47 [36–55]	0.096
**Right lung**
MPO positive cells, %	2.2 [1.2–9.2]	0.1 [0.1–0.1]^**^	0.4 [0.2–8.9]	8.0 [0.8–13.2]	**0.001**
AIF-1 positive cells, %	59 [39–86]	24 [24–37]^*^	41 [32–77]	59 [48–65]	**0.045**

*Data are expressed as median (quartiles)*.

*Comparisons are obtained with Kruskal–Wallis followed by Dunn's multiple comparison test*.

* *p < 0.05*,

** *p < 0.01 vs. Injury group*.

*MPO, Myeloperoxidase; AIF-1, Allograft inflammatory factor 1*.

*Bold values highlight significant differences*.

Left lungs from the FiCO_2_ group also showed the lowest presence of apoptotic cells as detected by the TUNEL assay (Supplementary Figure S2). Conversely, the lungs from the injury, low V_T_, and instrumental V_D_ showed a high prevalence of apoptotic cells within the lung parenchyma (Supplementary Figure S2).

### Protection of the Right Lung

**Figure 4:**
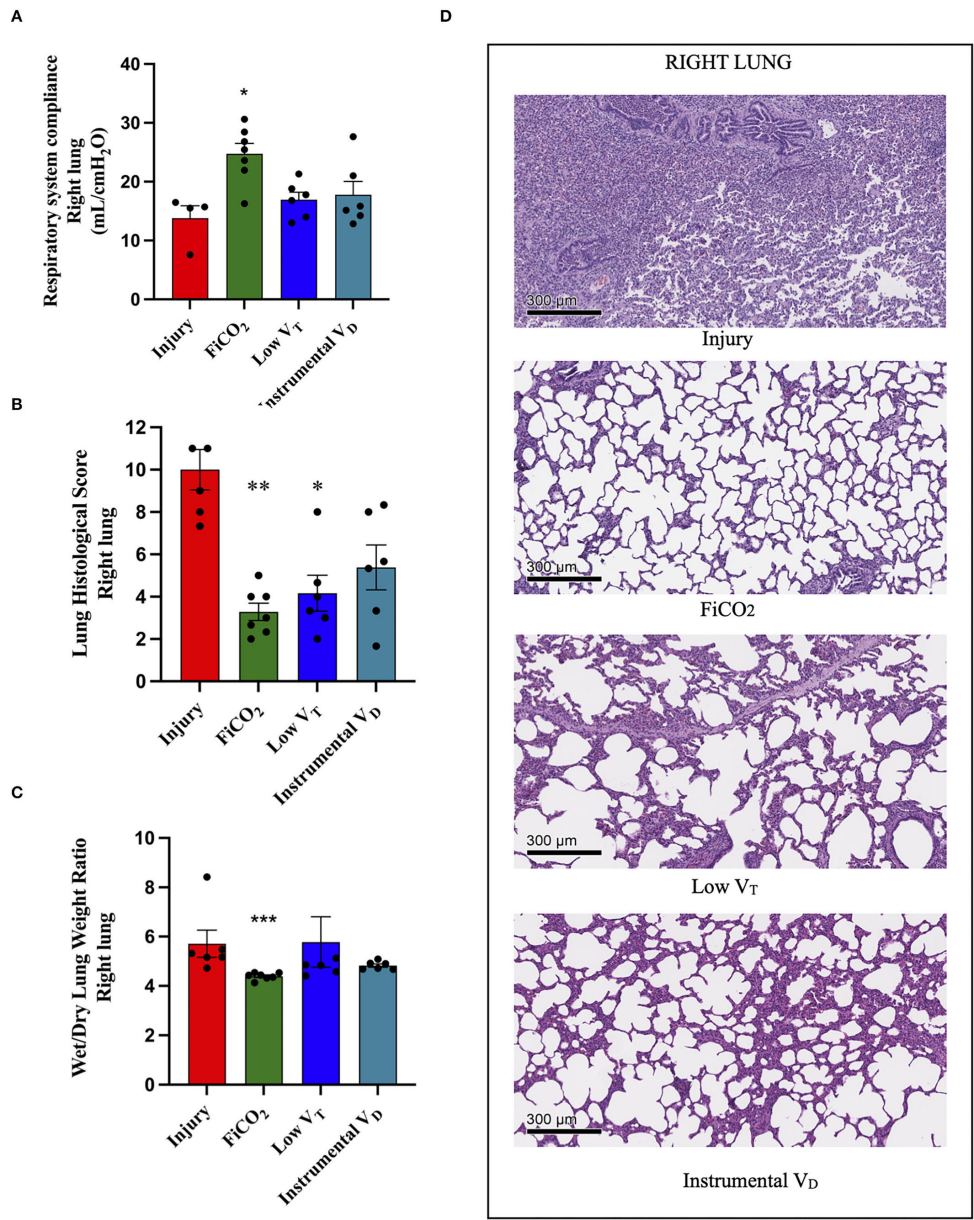
Right lung injury. Right-side respiratory system compliance at the end of the experiment **(A)**. Histological score of right lungs from each study group **(B)**. Wet-to-dry of right lungs **(C)**. Microscopic appearance of the lungs at the end of the experiment **(D)**. representative microphotographs of the right lungs from the four study groups (H&E, original magnification 100 ×). Data are expressed as scatter dot plots with mean ± SEM. Comparisons are obtained with ordinary one-way ANOVA or Kruskal–Wallis test for normally and non-normally distributed values, respectively, followed by Dunnett or Dunn's multiple comparison test. *p < 0.05, **p < 0.01 vs. Injury group, and *** *p* < 0.001 vs. Injury group.

The signs of injury in the right lung differed between groups (*p* = 0.021 for right-side compliance, *p* = 0.005 for histological score, *p* = 0.001 for wet-to-dry ratio). The right-side compliance was higher in the FiCO_2_ [25 (22 – 28)], while the other two hypercapnic groups did not differ from the injury group [17 (14 – 19) in low V_T_ *vs*. 16 (14 – 23) in instrumental V_D_ *vs*. 16 (10 – 16) in the injury] (Figure 4A). However, the histological score was lower both in the FiCO_2_ (3 ± 1) and in the low V_T_ groups (4 ± 2) as compared to the injury (10 ± 2) (Figure 4B). Like the right-side compliance, the wet-to-dry ratio was lower only in the FiCO_2_ group [4.4 (4.3–4.5) vs 4.8 (4.5–6.6) in the low V_T_, 4.8 (4.7–5.0) in the instrumental V_D_, 5.2 (5.1–6.2) in the injury] (Figure 4C).

Again, except for the right lung of the FiCO_2_ group – which showed nearly normal histologic appearance – various patterns of injury were observed in all the study groups at histological microscopic analysis. The right lungs of the injury group presented almost complete consolidation with a dense inflammatory infiltrate, composed of granulocytes, histiocytes, and lymphocytes, while right lung injury in the low V_T_ and instrumental V_D_ consisted in a mild macrophagic infiltrate with focal areas of emphysema (Figure 4D).

Inflammation in the right lung measured by immunohistochemistry was decreased only in the FiCO_2_ group, in which MPO-positive neutrophils were almost absent, while the low V_T_ and instrumental V_D_ groups showed similar or even higher levels of neutrophils compared to the injury group (Table 1 and Supplementary Figure S1).

### Distribution of Ventilation and Perfusion by EIT

**Figure 5:**
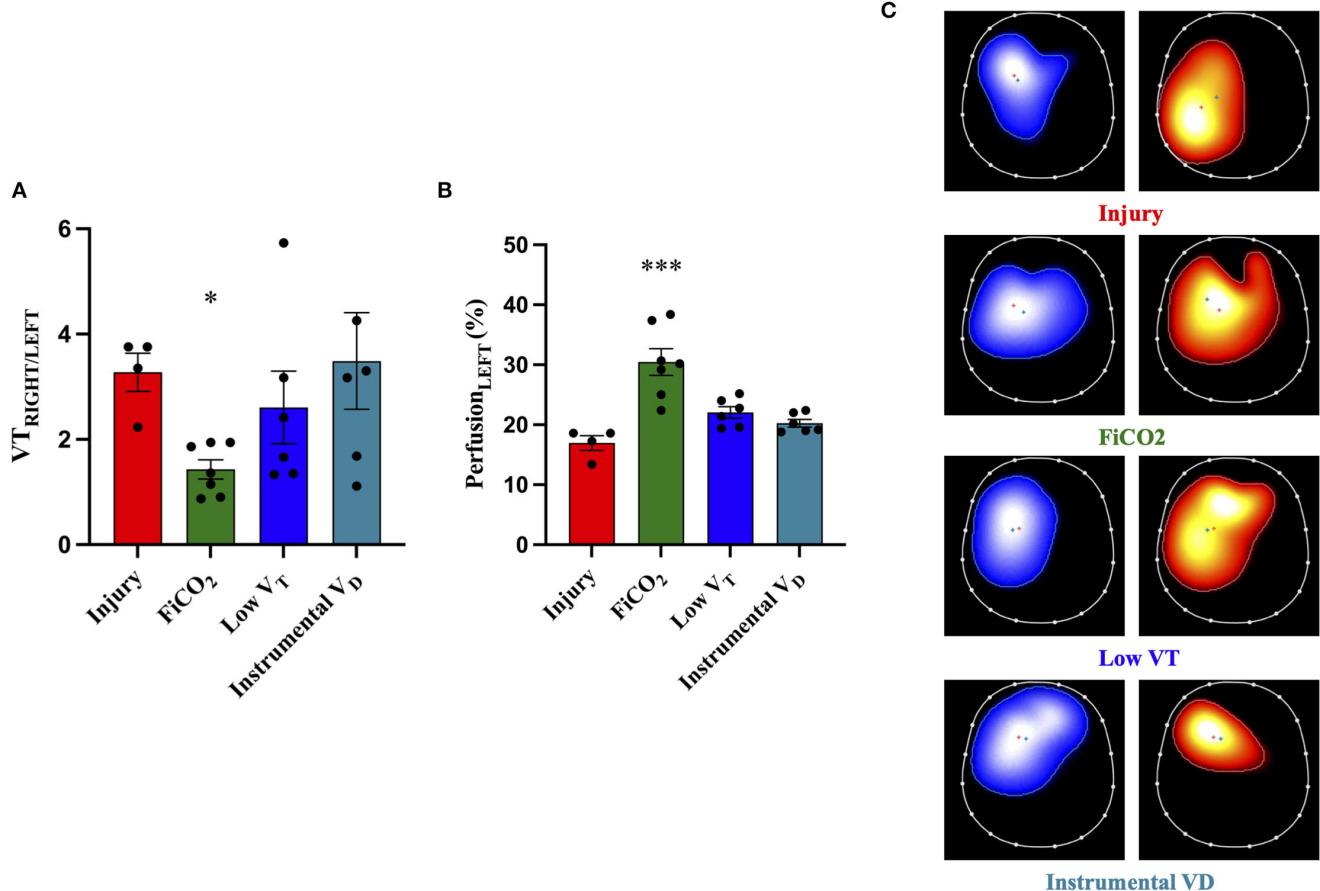
Distribution of ventilation and perfusion by EIT. The ratio between tidal volume distending the right and the left lung [V_TRIGHT/LEFT_, **(A)**] at 2 h after ligation of the left pulmonary artery shows significant imbalance in all the study groups, which was decreased only by FiCO_2_. The percentage of blood flow to the left lung [Perfusion_LEFT_, **(B)**] throughout the experiment (average between T2 and T48) was higher in the FiCO_2_ group compared to the other groups. Representative EIT images for ventilation (blue maps), perfusion (red maps), and distribution **(C)** showed increased ventilation and perfusion of the left lung in the FiCO_2_ group. Data are expressed as mean ± SEM. Comparisons are obtained with ordinary one-way ANOVA or Kruskal–Wallis test for normally and non-normally distributed values, respectively, followed by Dunnett or Dunn's multiple comparison test. *p < 0.05, ***p < 0.001 vs. Injury group.

The EIT analysis performed 2 h after pulmonary artery ligation (i.e., a time-point at which mechanisms of injury were already at play but lungs were not injured yet) showed that the ratio between the percentage of tidal volume to the right and the percentage of tidal volume to the left lung was significantly lower in the FiCO_2_ group as compared to all the other study groups (Figure 5A).

Regional perfusion measured by EIT throughout the study showed that the percentage of blood flow reaching the left ligated lung was higher in the group FiCO_2_ compared to the other groups (Figure 5B).

Representative EIT images showing the distribution of ventilation and perfusion in the four study groups are displayed in Figure 5C.

### Baseline

Before the start of the experiment (i.e., at baseline, measured before the ligation procedure with standard ventilation settings), there were no differences between the animals allocated to the four groups in terms of respiratory mechanics, gas exchange, and hemodynamics (Supplementary Table S2).

### Trends of Physiological Variables Over Time

**Figure 6:**
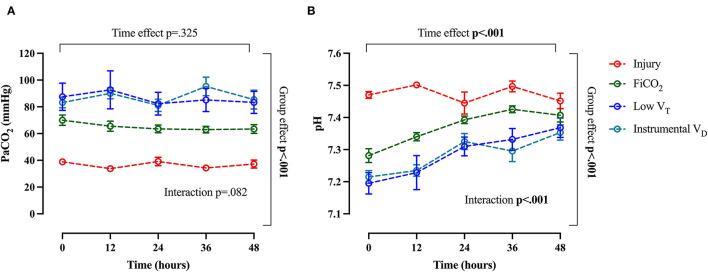
Trend of arterial CO_2_ and arterial pH throughout the study. Arterial pCO_2_ **(A)** and pH **(B)**. Data are expressed as mean ± SEM. Comparisons are obtained with a two-way ANOVA test for normally distributed values followed by Dunnett's multiple comparisons test.

**Figure 7:**
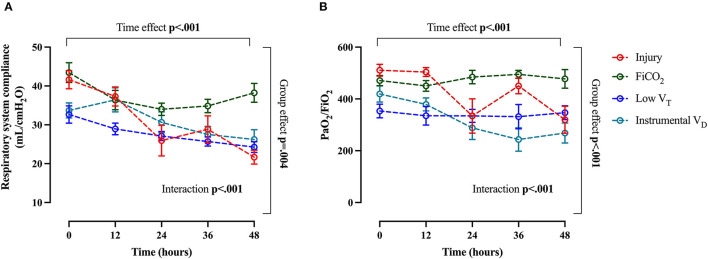
Main global markers of injury. Trend of respiratory system compliance **(A)** and PaO_2_/FiO_2_ **(B)** throughout the study. Data are expressed as mean ± SEM. Comparisons are obtained with two-way ANOVA test for normally distributed values followed by Dunnett's multiple comparisons test.

The course of arterial CO_2_ and arterial pH throughout the study are shown in Figures 6A,B. The evolution of injury through changes in respiratory system compliance and PaO_2_/FiO_2_ is shown in Figures 7A,B. The data collected for each variable at all time-points in the four study groups can be found in Supplementary Table S3 and confirm that the most differences appeared after 24 h.

## Discussion

The main findings of this study can be summarized as follows: The addition of 5% CO_2_ to inhaled gas confers full bilateral lung protection in experimental unilateral pulmonary artery ligation; hypercapnia is obtained by lowering the tidal volume and by increasing the instrumental dead space, instead, offers limited lung protection, if any, to the right perfused lung and fails to protect the left ligated lung. Mechanisms of lung protection by inhaled CO_2_ were confirmed in terms of reduced overdistension of the right lung and dampened bilateral lung inflammation. In this context, this study provides novel evidence of the additional role of increased regional perfusion reaching the left ligated lung (probably through the bronchial circulation) in preventing left lung injury.

This experimental study assessed the protection from VILI conferred by inhaled CO_2_ compared to hypercapnia induced by the low tidal volume and by the increased instrumental dead space in a model of unilateral pulmonary artery ligation. We confirmed that the protection of the left ligated lung is effectively achieved by addition of 5% CO_2_ to the inspired gas while the alternative methods used to induce hypercapnia were not effective. Our results confirm the mechanisms of injury for the left lung previously described as follows: Inflammation (12) and apoptosis triggered by alveolar hypocapnia (4), which could be effectively prevented by inhaled CO_2_. Interestingly, our results also showed that, in the left lung, plasmatic hypercapnia induced by the decreased tidal volume or the increased instrumental dead space did not prevent infiltration and activation of immune cells and did not prevent apoptosis. In contrast to adding inspired CO_2_, alveolar hypoventilation due to instrumental dead space might result in an uneven distribution of CO_2_ within the lungs, with a limited increase of CO_2_ content in the left lung due to the dilution with external gases and pulmonary artery perfusion block. A new finding is that inhalation of CO_2_, but not plasmatic hypercapnia obtained by the other two methods, increases blood flow to the left ligated lung. Our methods do not provide evidence for the source of this higher regional blood flow, but it is likely to derive from bronchial circulation (22). Indeed, bronchial perfusion increases in the presence of higher alveolar O_2_ and CO_2_ (23); both might have been obtained by inhaled CO_2_ through direct effect (for CO_2_) and higher regional ventilation of the left lung (for O_2_). Our observation suggests that the reduction of tissue hypoperfusion might be a novel mechanism underlying the protective effect of inhaled CO_2_ (8, 9, 24).

Concerning the right lung, protection was granted by addition of inhaled 5% CO_2_ – confirming the previous data (12) – but also by ventilation with low tidal volume. Plasmatic hypercapnia induced by the increased instrumental dead space, instead, was not effective. We have previously reported that hyperventilation and activation of inflammation are the key injurious mechanisms for the right non-ligated lung, which can be effectively prevented by inhaled CO_2_ (12). In the low V_T_ group, reduced over-distension and plasmatic hypercapnia seemed to protect the right non-ligated lung. However, in our model, right lung protection by low VT was inferior to inhaled CO_2_, as suggested. However, in our model, lung protection conferred by the low tidal volume was inferior compared to the inhaled CO_2_, as suggested by higher regional markers of inflammation, lower right-side respiratory system compliance, and higher wet-to-dry ratio. A reason for partial right lung protection by reduced V_T_ might be a lack of prevention of left ligated lung injury yielding organs cross-talk (25).

When we consider the global physiological consequences of lung injury in terms of impairment of oxygenation and respiratory mechanics, they once again confirm that only inhaled CO_2_ appears to confer effective protection from VILI in this model.

Experimental studies have demonstrated that therapeutic hypercapnia induced by inhaled CO_2_ is effective in attenuating lung injury in ARDS (15) and in protecting from VILI induced by high tidal volume ventilation (13, 14, 26). Regarding the role of hypercapnia induced by reduction of tidal volume (“permissive hypercapnia”), it has been difficult to separate the protective effect of hypercapnia *per se* from the established benefit due to the reduction of lung stress and strain (27). Clinical studies indicate that the permissive hypercapnia could improve clinical outcomes (28), but, at the same time, ARDS patients ventilated by a protective strategy developing severe hypercapnia are at higher risk of mortality (29). Experimental studies aimed at dissecting the protective effects of hypercapnia *vs*. those of low tidal volume have been scarce and led to conflicting results. Hypercapnia induced by reduced tidal volume and the respiratory rate has been shown to amplify inflammatory lung injury in experimental lipopolysaccharide-induced ARDS (30). On the contrary, ventilation with low tidal volumes and associated hypercapnia was proved protective in a model of surfactant depletion (31), although the protective effect seemed to depend mainly upon lower tidal volume (32). In contrast to adding inspired CO_2_, alveolar hypoventilation by low tidal volumes might result in an uneven distribution of CO_2_ within the lungs (33), and this could lead to failure to correct areas of alveolar hypocapnia. In conclusion, the reduced tidal volume ventilation with or without the permissive hypercapnia remains a cornerstone of ARDS treatment, but the evidence is growing on the potential role of inhaled CO_2_ as a complementary strategy leading to full lung protection.

This study has limitations. First, the results partially overlap with our previous work (12); however, we present alternative methods to obtain hypercapnia, as well as new data on regional lung perfusion and apoptosis, which increased our understanding of pathophysiological mechanisms. Second, we applied pragmatic methods to obtain plasmatic hypercapnia using a fixed value for low tidal volume and adding instrumental dead space targeted to a target CO_2_ level. Other methods might have led to different results; however, our methods reflected values normally used in clinical practice and increase potential clinical translation. Third, alveolar hypocapnia, which is a key mechanism of injury in our model, was not measured, so we can only hypothesize its role to explain the differences between groups. Finally, EIT is a technique with limitations, including imaging limited to a portion of the lung and the relative nature of the measures of regional ventilation and perfusion.

## Conclusion

This study shows that inhaled CO_2_ allows more effective bilateral lung protection compared to plasmatic hypercapnia induced by low tidal volume and additional instrumental dead space in a model of left pulmonary artery ligation. Further studies are needed to understand whether a protective strategy combining low tidal volume and inhaled CO_2_ might be beneficial in patients with large perfusion defects (e.g., ARDS with high dead space fraction).

## Data Availability Statement

The raw data supporting the conclusions of this article will be made available by the authors, without undue reservation.

## Ethics Statement

The animal study was reviewed and approved by Italian Ministry of Health (protocol n. 543/2018-PR).

## Author Contributions

ES, AP, and TM conceived, planned, carried out the experiments, interpreted the results, and wrote the manuscript. GL, AD, FD, EG, GD, AC, GG, VF, OB, MB, and CL carried out the experiments and collected the results. VV processed experimental data. OB and MB helped in the implementation of the experiments. GL, VV, and SF collected and processed biological samples and analyzed the results. LR, SF, and SG provided critical feedback, helped shape the research, analysis, and manuscript. All authors contributed to the article and approved the submitted version.

## Funding

The work was funded by Ricerca Finalizzata 2016 from the Italian Ministry of Health, Rome, Italy to TM, project GR-2016-02362428 and Ricerca Corrente 2021 from Ospedale Maggiore Policlinico of Milan, Italy to AP.

## Conflict of Interest

AP reports personal fess from Baxter, Maquet, Boehringer Ingelheim and Xenios outside the submitted work. TM reports personal fees from Drager, Fisher and Paykel, Hamilton and BBraun outside the submitted work. The remaining authors declare that the research was conducted in the absence of any commercial or financial relationships that could be construed as a potential conflict of interest.

## Publisher's Note

All claims expressed in this article are solely those of the authors and do not necessarily represent those of their affiliated organizations, or those of the publisher, the editors and the reviewers. Any product that may be evaluated in this article, or claim that may be made by its manufacturer, is not guaranteed or endorsed by the publisher.

## References

[1] Zapol WM Jones R. Vascular components of ARDS. Clinical pulmonary hemodynamics and morphology. Am Rev Respir Dis. (1987) 136:471–4. 10.1164/ajrccm/136.2.471 3619211

[2] Patel BV Arachchillage DJ Ridge CA Bianchi P Doyle JF Garfield B. Pulmonary angiopathy in severe COVID-19: physiologic, imaging, and hematologic observations. Am J Respir Crit Care Med. (2020) 202:690–9. 10.1164/rccm.202004-1412OC 32667207 PMC7462405

[3] Laffey JG Engelberts D Kavanagh BP. Injurious effects of hypocapnic alkalosis in the isolated lung. Am J Respir Crit Care Med. (2000) 162:399–405. 10.1164/ajrccm.162.2.9911026 10934060

[4] Kiefmann M Tank S Keller P Bornchen C Rinnenthal JL Tritt MO. IDH3 mediates apoptosis of alveolar epithelial cells type 2 due to mitochondrial Ca(2+) uptake during hypocapnia. Cell Death Dis. (2017) 8:e3005. 10.1038/cddis.2017.403 28837149 PMC5596584

[5] Tsang JY Lamm WJ Swenson ER. Regional CO2 tension quantitatively mediates homeostatic redistribution of ventilation following acute pulmonary thromboembolism in pigs. J Appl Physiol (1985). (2009) 107:755–62. 10.1152/japplphysiol.00245.2009 19608933

[6] Cambiaghi B Vasques F Morer O Ritter C Mauri T Kunze-Szikszay N. Effects of regional perfusion block in healthy and injured lungs. Intensive Care Med Exp. (2017) 5:46. 10.1186/s40635-017-0161-2 29030751 PMC5640557

[7] Langer T Castagna V Brusatori S Santini A Mauri T Zanella A. Short-term physiologic consequences of regional pulmonary vascular occlusion in pigs. Anesthesiology. (2019) 131:336–43. 10.1097/ALN.0000000000002735 31094756

[8] Tojo K Nagamine Y Yazawa T Mihara T Baba Y Ota S. Atelectasis causes alveolar hypoxia-induced inflammation during uneven mechanical ventilation in rats. Intensive Care Med Exp. (2015) 3:56. 10.1186/s40635-015-0056-z 26215820 PMC4480346

[9] Kawabata Y Shimizu Y Hoshi E Ikeya T Kurashima K Takayanagi N. Acute ischaemic lung injury due to pulmonary vascular obstruction. Histopathology. (2016) 69:647–54. 10.1111/his.12979 27040641

[10] Gattinoni L Protti A Caironi P Carlesso E. Ventilator-induced lung injury: the anatomical and physiological framework. Crit Care Med. (2010) 38:S539–548. 10.1097/CCM.0b013e3181f1fcf7 21164395

[11] Henderson WR Chen L Amato MBP Brochard LJ. Fifty years of research in ARDS. Respiratory mechanics in acute respiratory distress syndrome. Am J Respir Crit Care Med. (2017) 196:822–33. 10.1164/rccm.201612-2495CI 28306327

[12] Marongiu I Spinelli E Scotti E Mazzucco A Wang YM Manesso L. Addition of 5% CO2 to inspiratory gas prevents lung injury in an experimental model of pulmonary artery ligation. Am J Respir Crit Care Med. (2021) 204:933–42. 10.1164/rccm.202101-0122OC 34252009 PMC8534619

[13] Broccard AF Hotchkiss JR Vannay C Markert M Sauty A Feihl F. Protective effects of hypercapnic acidosis on ventilator-induced lung injury. Am J Respir Crit Care Med. (2001) 164:802–6. 10.1164/ajrccm.164.5.2007060 11549536

[14] Sinclair SE Kregenow DA Lamm WJ Starr IR Chi EY Hlastala MP. Hypercapnic acidosis is protective in an *in vivo* model of ventilator-induced lung injury. Am J Respir Crit Care Med. (2002) 166:403–8. 10.1164/rccm.200112-117OC 12153979

[15] Laffey JG Honan D Hopkins N Hyvelin JM Boylan JF McLoughlin P. Hypercapnic acidosis attenuates endotoxin-induced acute lung injury. Am J Respir Crit Care Med. (2004) 169:46–56. 10.1164/rccm.200205-394OC 12958048

[16] Severinghaus JW Swenson EW Finley TN Lategola MT Williams J. Unilateral hypoventilation produced in dogs by occluding one pulmonary artery. J Appl Physiol. (1961) 16:53–60. 10.1152/jappl.1961.16.1.53 13750428

[17] Shepard JW Jr Hauer D Miyai K Moser KM. Lamellar body depletion in dogs undergoing pulmonary artery occlusion. J Clin Invest. (1980) 66:36–42. 10.1172/JCI109832 6772668 PMC371502

[18] Fan E Del Sorbo L Goligher EC Hodgson CL Munshi L Walkey AJ. American Thoracic Society ESoICM, Society of Critical Care M. An Official American Thoracic Society/European Society of Intensive Care Medicine/Society of Critical Care Medicine Clinical Practice Guideline: Mechanical Ventilation in Adult Patients with Acute Respiratory Distress Syndrome. Am J Respir Crit Care Med. (2017) 195:1253–63. 10.1164/rccm.201703-0548ST 28459336

[19] Spinelli E Kircher M Stender B Ottaviani I Basile MC Marongiu I. Unmatched ventilation and perfusion measured by electrical impedance tomography predicts the outcome of ARDS. Crit Care. (2021) 25:192. 10.1186/s13054-021-03615-4 34082795 PMC8173510

[20] Meyerholz DK Lambertz AM Reznikov LR Ofori-Amanfo GK Karp PH McCray PB Jr. Immunohistochemical detection of markers for translational studies of lung disease in pigs and humans. Toxicol Pathol. (2016) 44:434–41. 10.1177/0192623315609691 26511846 PMC4805467

[21] Faversani A Vaira V Moro GP Tosi D Lopergolo A Schultz DC. Survivin family proteins as novel molecular determinants of doxorubicin resistance in organotypic human breast tumors. Breast Cancer Res. (2014) 16:R55. 10.1186/bcr3666 24886669 PMC4076638

[22] Jindal SK Lakshminarayan S Kirk W Butler J. Acute increase in anastomotic bronchial blood flow after pulmonary arterial obstruction. J Appl Physiol Respir Environ Exerc Physiol. (1984) 57:424–8. 10.1152/jappl.1984.57.2.424 6469812

[23] Baile EM Pare PD. Response of the bronchial circulation to acute hypoxemia and hypercarbia in the dog. J Appl Physiol Respir Environ Exerc Physiol. (1983) 55:1474–9. 10.1152/jappl.1983.55.5.1474 6417080

[24] Frohlich S Boylan J McLoughlin P. Hypoxia-induced inflammation in the lung: a potential therapeutic target in acute lung injury? Am J Respir Cell Mol Biol. (2013) 48:271–9. 10.1165/rcmb.2012-0137TR 23087053

[25] Setzer F Schmidt B Hueter L Schwarzkopf K Sanger J Schreiber T. Characterization of the seven-day course of pulmonary response following unilateral lung acid injury in rats. PLoS ONE. (2018) 13:e0198440. 10.1371/journal.pone.0198440 29864150 PMC5986146

[26] Laffey JG Engelberts D Duggan M Veldhuizen R Lewis JF Kavanagh BP. Carbon dioxide attenuates pulmonary impairment resulting from hyperventilation. Crit Care Med. (2003) 31:2634–40. 10.1097/01.CCM.0000089646.52395.BA 14605535

[27] Acute Respiratory Distress Syndrome N Brower RG Matthay MA Morris A Schoenfeld D Thompson BT. Ventilation with lower tidal volumes as compared with traditional tidal volumes for acute lung injury and the acute respiratory distress syndrome. N Engl J Med. (2000) 342:1301–8. 10.1056/NEJM200005043421801 10793162

[28] Hickling KG Henderson SJ Jackson R. Low mortality associated with low volume pressure limited ventilation with permissive hypercapnia in severe adult respiratory distress syndrome. Intensive Care Med. (1990) 16:372–7. 10.1007/BF01735174 2246418

[29] Nin N Muriel A Penuelas O Brochard L Lorente JA Ferguson ND. Severe hypercapnia and outcome of mechanically ventilated patients with moderate or severe acute respiratory distress syndrome. Intensive Care Med. (2017) 43:200–8. 10.1007/s00134-016-4611-1 28108768 PMC5630225

[30] Lang JD Figueroa M Sanders KD Aslan M Liu Y Chumley P. Hypercapnia via reduced rate and tidal volume contributes to lipopolysaccharide-induced lung injury. Am J Respir Crit Care Med. (2005) 171:147–57. 10.1164/rccm.200302-305OC 15477499

[31] Fuchs H Mendler MR Scharnbeck D Ebsen M Hummler HD. Very low tidal volume ventilation with associated hypercapnia–effects on lung injury in a model for acute respiratory distress syndrome. PLoS ONE. (2011) 6:e23816. 10.1371/journal.pone.0023816 21886825 PMC3158784

[32] Hummler HD Banke K Wolfson MR Buonocore G Ebsen M Bernhard W. The effects of lung protective ventilation or hypercapnic acidosis on gas exchange and lung injury in surfactant deficient rabbits. PLoS ONE. (2016) 11:e0147807. 10.1371/journal.pone.0147807 26840779 PMC4739580

[33] Swenson ER. Therapeutic hypercapnic acidosis: pushing the envelope. Am J Respir Crit Care Med. (2004) 169:8–9. 10.1164/rccm.2310008 14695104

