# Supplementary material for: Inhaled CO2 vs. Hypercapnia Obtained by Low Tidal Volume or Instrumental Dead Space in Unilateral Pulmonary Artery Ligation: Any Difference for Lung Protection?
Source: Front Med (Lausanne). 2022 May 20;9:901809. doi: 10.3389/fmed.2022.901809 (PMC9163369; doi:10.3389/fmed.2022.901809)

**Inhaled CO_2_ versus hypercapnia obtained by low tidal volume or instrumental dead space in unilateral pulmonary artery ligation**

***Any difference for lung protection?***

Elena Spinelli, Antonio Pesenti, Gianluca Lopez, Anna Damia, Francesco Damarco, Erica Garbelli, Gaia Dal Santo, Alessio Caccioppola, Giorgio Giudici, Virginia Figgiaconi, Osvaldo Biancolilli, Michele Battistin, Caterina Lonati, Valentina Vaira, Lorenzo Rosso, Stefano Ferrero, Stefano Gatti, Tommaso Mauri

**Online data supplement**

**Appendix I. Supplementary methods**

The study was approved by the Italian Ministry of Health (protocol n. 543/2018-PR) and conducted according to the European Directive 2010/63/EU on the protection of animals used for scientific purposes and Italian legislative decree 26/2014. Approval by the Institutional Animal Care Committee was obtained before starting the experiments.

**Animal preparation**. In compliance with local recommendations, pigs arrived at the experimental facility the day before the start of the study and fasted overnight with free access to water. Sedation was administered by intramuscular injection of medetomidine 0.025 mg/kg and tiletamine/zolazepam 5 mg/kg. Then, an auricular vein was cannulated and, after administration of cefazoline 1 g and tramadol 50 mg, continuous intravenous (IV) infusion of propofol was titrated to maintain the animal on spontaneous breathing and SpO_2_ 100% while on additional oxygen via face mask. Surgical tracheostomy was performed in the supine position under additional local anesthesia (lidocaine 2%). After endotracheal tube was inserted through the tracheostomy and fixed, mechanical ventilation was started with baseline settings and general anesthesia and neuromuscular blockade were maintained by IV propofol 5-10 mg/kg/h, medetomidine 2.5-10.0 μg/kg/h and pancuronium bromide 0.3-0.5 mg/kg/h. During the whole experiment, oxygen saturation, heart rate, invasive blood pressure, central venous pressure, pulmonary artery pressure and ventilatory waveforms were monitored. Depth of anesthesia was adjusted to ensure no sign of distress, such as unexplained tachycardia, arterial hypertension and/or horripilation. External warming was provided when core body temperature fell below 37.5°C, as well as external cooling when core body temperature rose above 40°C.

Ringer lactate was administered at 100 ml/h during surgery and reduced to 50 ml/h along the whole study, aiming at a zero fluid balance.

Cefazoline 1 g IV and tramadol 50 mg IV were administered every 12 hours. Low molecular weight heparin 2000 IU was administered subcutaneously once per day.

**Instrumentation.** Vascular accesses were obtained by surgical exposure. An arterial catheter (Seldicath®, 5 Fr 8 cm, Prodimed, France) was inserted in the right common carotid artery. A three-lumen central venous catheter (Arrow®, 7 Fr, Teleflex, Ireland) and a pulmonary artery catheter (Swan Ganz®, 5 Fr, Edwards, USA) were introduced in the right external jugular vein. Positioning of pulmonary artery catheter was guided by visualization of pulmonary artery and wedge pressures. An esophageal balloon catheter (Cooper Surgical, USA) was inserted and inflated with the recommended volume of air. Correct positioning and calibration were confirmed by the standard occlusion test with external compressions.

Left pulmonary artery ligation. Left pulmonary artery was chosen for the less invasive surgical approach if compared to the right side. Left pulmonary artery ligation was performed as follows: at the level of the 4th or 5th intercostal space, a 10 cm left thoracotomy was performed with the animal in the right lateral position. After the retraction of the left azygos vein, the main left pulmonary artery was isolated and progressively (5 minutes) occluded and then ligated with a non-absorbable 2/0 silk suture thread. A chest tube was placed, and the chest was closed with multi-strata suture. Finally, to evacuate residual air from the pleural cavity, the chest tube was removed during a recruitment maneuver.

**Study measurements.** Pulsossimetry, heart rate, invasive arterial pressure and pulmonary artery pressure were continuously monitored. End-tidal CO_2_ was measured through a capnometer (Respironics NM3®, Philips, The Netherlands). A 16 electrodes EIT belt was positioned around the swine's chest, connected to an EIT monitor (PulmoVista® 500, Dräger, Lübeck, Germany) and left in place throughout the study period.

Bronchial suctioning followed by a recruitment maneuver (pressure controlled mode, 45 cmH2O, I:E=1:1, respiratory rate 10 bpm, for 1 minute/10 breaths) were performed 20 minutes before each timepoint.

The variables collected were:

- Respiratory mechanics: airway peak pressure (Ppeak), plateau pressure (Pplat) by a 3-second inspiratory pause, mean airway pressure (Pmean), total PEEP (PEEPtot) by a 3-second expiratory pause, V_T_, change between inspiratory and expiratory esophageal pressure (ΔPes). From these, driving pressure (DP) was calculated as Pplat –PEEPtot and driving transpulmonary pressure as DP- ΔPes; static respiratory system compliance (C_RS_) was calculated as V_T_/DP; lung compliance (C_L_) as V_T_/ΔPL; chest wall elastance (C_CW_) as V_T_/ΔPes

- Hemodynamics: systolic, diastolic and mean arterial pressures (SAP, DAP, MAP); systolic, diastolic and mean pulmonary artery pressures (PAPS, PAPD, PAPM); wedge pressure (WP) at end expiration; cardiac output (CO) via thermodilution technique (Vigilance, Baxter Edwards Critical Care, Edwards E6 Lifesciences, USA); heart rate (HR); central venous pressure (CVP) at end expiration, mixed venous oxygen saturation (SvO_2_).

- Gas exchange: arterial pH, PaCO_2_, PaO_2_.

- End-tidal CO_2_

- EIT data: EIT data were recorded at 50 Hz and stored for offline analysis. EIT ventilation and perfusion maps were obtained by dedicated software (Dräger EIT Data Analysis Tool 6.3, Lübeck, Germany). We split the EIT images into two same size regions of interests on the horizontal axis from halfway left and right, roughly corresponding to the right and left lungs. For ventilation maps we averaged values over 8 consecutive respiratory cycles.

Euthanasia, autopsy, histology, immunohistochemistry and apoptosis detection. At the end of the experiment, animals were euthanized (by intravenous injection of potassium chloride 40 mEq under deep sedation) and underwent autopsy for collection of histological samples. Lungs were promptly excised en bloc via sternotomy and the complete occlusion of the left pulmonary artery was confirmed by direct visualization. For each pig’s lungs twelve representative tissue samples (2 from upper, 2 from medial and 2 from inferior lobes for each side) of 1 cm^3^ volume were collected: 6 were stored in formaldehyde to undergo histological examination and 6 were used to calculate wet to dry ratio. Two pathologists blinded to study group and to the original location of the sample conducted the examination. Right and left histological score of the lungs was calculated from the six samples per animal stored in formaldehyde (3 for each side). Ten main histological alterations were evaluated: emphysematous change, interstitial congestion, alveolar hemorrhage, alveolar neutrophil infiltration, alveolar macrophage proliferation, alveolar type II pneumocytes proliferation, interstitial lymphocytes proliferation, interstitial thickening, hyaline membrane formation and organization of alveolar exudate. Each alteration was scored from 0 (absent) to 3 (severe) in each sample (ten values from zero to 3 for each sample); then, the histological score of that sample was calculated as the sum of the ten values; finally, the regional histological score of the lungs was calculated as the average value of the scores of the 3 samples for each side (range: 0 to 30).

Samples for the wet to dry calculation were weighed before and after being dried for 24 hours at 50°C in an oven. The average value of the scores of the 3 samples for each side was then calculated.

Formalin-fixed and paraffin-embedded blocks of lung tissue samples of the four groups underwent quantitative immunohistochemistry analysis to identify the percentage of cells positive for myeloperoxidase (MPO, for neutrophils) and ionized calcium-binding adaptor 1 (IBA-1, for macrophages). The percentage of positive cells was obtained in samples from medial lobes of each lung. Stainings were performed in a Dako automated instrument and then slides were digitally scanned using Aperio (Leica Microsystems, Milan, Italy). Then, the percentage of positive cells was calculated using the ImageScope console and optimized algorithms (Leica Microsystems), as previously described (1).

The ApopTag Peroxidase In Situ Apoptosis Detection Kit (Tunel assay; Merck KGaA, Germany) was used as previously described (2) for apoptosis detection on the left lungs of representative animals (n = 2) for each study group.

*Sample size calculation.* An effect size of 0.75 was calculated based on our previous results (1), and in particular on a mean histological score of 11 in the control group and of 5 in the FiCO2 group. We hypothesized an intermediate score of 7 for the other two groups, for which preliminary results were not available, and a standard deviation of 3 within each group. The effect size and the sample size was computed using G*Power Software (ver 3.1.9.2; Heinrich-Heine-Universität Düsseldorf, Düsseldorf, Germany) for an Anova test on 4 groups, with a power of 0.8 and an alpha of 0.05.

**References**

1. Marongiu I, Spinelli E, Scotti E, Mazzucco A, Wang YM, Manesso L, Colussi G, Biancolilli O, Battistin M, Langer T, Roma F, Lopez G, Lonati C, Vaira V, Rosso L, Ferrero S, Gatti S, Zanella A, Pesenti A, Mauri T. Addition of 5% CO2 to Inspiratory Gas Prevents Lung Injury in an Experimental Model of Pulmonary Artery Ligation. *Am J Respir Crit Care Med* 2021; 204: 933-942.

2. Faversani A, Vaira V, Moro GP, Tosi D, Lopergolo A, Schultz DC, Rivadeneira D, Altieri DC, Bosari S. Survivin family proteins as novel molecular determinants of doxorubicin resistance in organotypic human breast tumors. *Breast Cancer Res* 2014; 16: R55.

**Appendix II. Supplementary Figures**

**Figure S1. Characterization of the lung immune cell infiltrates in the different groups by**

**immunohistochemistry**. Representative images of the MPO-positive infiltrates in the left and right lungs of pigs from the four different study groups. Bars (magnification 10x).

**Figure S2. Detection of apoptotic cells in the left lung by TUNEL assay.** Representative images of Apotag staining in the left lungs from the four different study groups. Brown staining corresponds to apoptotic cells.

**Table S1. Physiological characteristics of the study groups at the end of the experiment (T48).**

|  | **Injury**  **(n=6)** | **FiCO_2_**  **(n=7)** | **Low V_T_**  **(n=6)** | **InstrumentalV_D_**  **(n=6)** | **ANOVA**  **p value** |
| --- | --- | --- | --- | --- | --- |
| **Respiratory mechanics** | | | | | |
| **Peak Pressure (cmH_2_O)** | 32 [28 – 37] | 20 [18 – 22]* | 16 [15 – 20] ** | 27 [22 – 33] | **<.001** |
| **Plateau Pressure (cmH_2_O)** | 23 [22 – 25] | 14 [13 – 16]** | 14 [13 – 17]** | 19 [16 – 23] | **.002** |
| **Mean Airway Pressure (cmH_2_O)** | 13 ± 2 | 9 ± 1** | 8 ± 1** | 10 ± 2 | **.002** |
| **Driving Pressure (cmH_2_O)** | 18 [17 – 20] | 8 [8 – 10]** | 9 [8 – 11]** | 14 [11 – 18] | **<.001** |
| **Respiratory System Compliance (ml/cmH_2_O)** | 22 ± 4 | 38 ± 6** | 24 ± 3 | 26 ± 6 | **.003** |
| **Lung Compliance (ml/cmH_2_O)** | 29 ± 8 | 79 ± 26** | 37 ± 9 | 44 ± 16 | **.003** |
| **Chest wall compliance (ml/cmH_2_O)** | 94 ± 27 | 80 ± 14 | 85 ± 35 | 70 ± 11 | .413 |
| **Arterial Blood Gas Analysis Data** | | | | | |
| **PaO_2_/FiO_2_ (mmHg)** | 354 [202 – 417] | 520 [384 – 542] | 381 [171 – 390] | 265 [186 – 329] | **.017** |
| **PaCO_2_ (mmHg)** | 37 ± 7 | 63 ± 9 | 83 ± 20** | 85 ± 17*** | **<.001** |
| **pH** | 7.47 [7.42 – 7.49] | 7.42 [7.40 – 7.45] | 7.37 [7.29 – 7.44] | 7.38 [7.32 –7.39]* | **.030** |
| **HCO_3_^-^ (mmol/L)** | 25.4 ± 2.8 | 37.8 ± 2.4 | 45.6 ± 3.4*** | 45.3 ± 3.5*** | **<.001** |
| **EtCO_2_ (mmHg)** | 28 ± 4 | 67 ± 6 | 81 ± 17** | 81 ± 15** | **.002** |
| **Hemodynamics** | | | | | |
| **Systolic Arterial Pressure (mmHg)** | 118 ± 7 | 107 ± 10 | 110 ± 8 | 120 ± 12 | .152 |
| **Diastolic Arterial Pressure (mmHg)** | 81 ± 9 | 63 ± 13 | 63 ± 12 | 63 ± 13 | .072 |
| **Mean Arterial Pressure (mmHg)** | 100 ± 8 | 86 ± 13 | 86 ± 10 | 78 ± 26 | .102 |
| **Systolic Pulmonary Artery Pressure (mmHg)** | 39 ± 6 | 31 ± 4* | 31 ± 4 | 34 ± 4 | **.044** |
| **Diastolic Pulmonary Artery Pressure (mmHg)** | 20 ± 7 | 15 ± 4 | 16 ± 3 | 18 ± 5 | .375 |
| **Mean Pulmonary Artery Pressure (mmHg)** | 30 ± 6 | 23 ± 3 | 24 ± 3 | 25 ± 5 | .109 |
| **Pulmonary Vascular Resistance (dyne/s/cm^-5^)** | 343 ± 73 | 248 ± 27 | 233 ± 38 | 277 ± 64 | .095 |
| **Wedge Pressure (mmHg)** | 11 ± 3 | 8 ± 1 | 9 ± 2 | 8 ± 3 | .227 |
| **Cardiac Output (L/min)** | 4.4 ± 0.8 | 5.1 ± 1.1 | 5.8 ± 0.8 | 5.2 ± 1.3 | .171 |
| **Heart Rate (bpm)** | 98 ± 15 | 95 ± 21 | 109 ± 23 | 119 ± 44 | .590 |
| **Fluid Balance (ml)** | -65 [-559 – 348] | -450 [-830–4000] | 17 [-720 – 379] | -75 [-401 – 63] | .992 |

Data are expressed as mean ± standard deviation (normally distributed values) or median [quartiles] (non-normally distributed values).
_a_Comparisons are obtained with one-way ANOVA or Kruskal-Wallis test for normally and non-normally distributed values respectively followed by Dunnett or Dunn’s multiple comparison tests as appropriate. *p<0.05, **p<0.01 and ***p<0.001 vs Injury group.

Abbreviations: PaO2/FiO2, partial pressure of oxygen / inspired fraction of oxygen; PaCO2, partial pressure of carbon dioxide; EtCO2, end-tidal CO2.

**Table S2. Physiological characteristics of the study groups at Baseline: after animal preparation and instrumentation and before surgical ligation of the left pulmonary artery.**

|  | **Injury**  **(n=6)** | **FiCO_2_**  **(n=7)** | **Low V_T_**  **(n=6)** | **Instrumental V_D_**  **(n=6)** | **p value** |
| --- | --- | --- | --- | --- | --- |
| **Respiratory mechanics** | | | | | |
| **Plateau Pressure (cmH_2_O)** | 14 [13 - 15] | 12 [12 – 14] | 14 [13 – 14] | 14 [13 – 15] | .312 |
| **Mean Airway Pressure (cmH_2_O)** | 10 [9 – 10] | 9 [8 – 9] | 9 [9 – 10] | 9 [9 – 10] | .122 |
| **Driving Pressure (cmH_2_O)** | 9 [8 – 10] | 7 [6 – 8] | 9 [8 – 9] | 9 [8 – 10] | .101 |
| **Respiratory System Compliance (ml/cmH_2_O)** | 46 ± 5 | 48 ± 7 | 42 ± 5 | 42 ± 8 | .265 |
| **Arterial Blood Gas Analysis Data** | | | | | |
| **PaO_2_/FiO_2_ (mmHg)** | 527 ± 30 | 539 ± 22 | 517 ± 42 | 523 ± 54 | .740 |
| **PaCO_2_ (mmHg)** | 37 ± 6 | 36 ± 5 | 39 ± 3 | 39 ± 4 | .399 |
| **pH** | 7.49 ± 0.03 | 7.51 ± 0.07 | 7.48 ± 0.01 | 7.49 ± 0.05 | .662 |
| **HCO_3_^-^ (mmol/L)** | 27.3 ± 2.1 | 28.6 ± 2.3 | 27.6 ± 1.5 | 29.0 ± 1.7 | .334 |
| **Hemodynamics** | | | | | |
| **Mean Arterial Pressure (mmHg)** | 105 ± 23 | 100 ± 16 | 118 ± 15 | 110 ± 8 | .197 |
| **Heart Rate (bpm)** | 101 ± 15 | 96 ± 8 | 96 ± 10 | 88 ± 10 | .369 |
|  |  |  |  |  |  |
| **Mean Pulmonary Artery Pressure (mmHg)** | 18 [15 – 19] | 18 [17 – 19] | 18 [16 – 20] | 17 [14 – 18] | .397 |
| **Cardiac Output (L/min)** | 4.3 [3.9 – 4.7] | 3.9 [3.8 – 4.5] | 3.9 [3.3 – 4.3] | 3.8 [3.2 – 3.9] | .178 |

Data are expressed as mean ± standard deviation (normally distributed values) or median [quartiles] (non-normally distributed values). Comparisons are obtained with one-way ANOVA or Kruskal-Wallis test for normally and non-normally distributed values respectively followed by Dunnett or Dunn’s multiple comparison test.

Abbreviations: PaO2/FiO2, partial pressure of oxygen / inspired fraction of oxygen; PaCO2, partial pressure of carbon dioxide.

**Table S3. Trends of physiological variables during the study**

|  | **T2** | **T12** | **T24** | **T36** | **T48** | **Time** | **Group** | **Time*Group** |
| --- | --- | --- | --- | --- | --- | --- | --- | --- |
| **Respiratory Mechanics** | | | | | | | | |
| **Peak Pressure (cmH_2_O)** |  | | | | | | | |
| Injury (n=6) | 22 ± 3 | 22 ± 3 | 32 ± 7 | 28 ± 4 | 39 ± 8 | **<0.001** | **<0.001** | **<0.001** |
| FiCO2 (n=7) | 19 ± 3 | 21 ± 3 | 21 ± 3* | 21 ± 3* | 20 ± 3* |  |  |  |
| Low V_T_ (n=6) | 15 ± 2* | 17 ± 3* | 17 ± 2** | 17 ± 3** | 18 ± 4** |  |  |  |
| Instrumental V_D_ (n=6) | 21 ± 3 | 21 ± 3 | 25 ± 6 | 26 ± 5 | 28 ± 6 |  |  |  |
| **Plateau Pressure (cmH_2_O)** |  | | | | | | | |
| Injury (n=6) | 15 ± 2 | 16 ± 2 | 22 ± 5 | 19 ± 3 | 24 ± 4 | **<0.001** | **<0.001** | **<0.001** |
| FiCO2 (n=7) | 14 ± 2 | 15 ± 2 | 15 ± 2 | 15 ± 1* | 14 ± 1** |  |  |  |
| Low V_T_ (n=6) | 12 ± 1* | 13 ± 1* | 13 ± 1* | 14 ± 3* | 15 ± 3** |  |  |  |
| Instrumental V_D_ (n=6) | 16 ± 2 | 15 ± 2 | 17 ± 3 | 18 ± 3 | 19 ± 4 |  |  |  |
| **Mean Airway Pressure (cmH_2_O)** |  | | | | | | | |
| Injury (n=6) | 10 ± 1 | 10 ± 1 | 11 ± 2 | 11 ± 1 | 13 ± 2 | **<0.001** | **<0.001** | **<0.001** |
| FiCO2 (n=7) | 9 ± 1 | 9 ± 1 | 10 ± 1 | 9 ± 1* | 9 ± 1* |  |  |  |
| Low V_T_ (n=6) | 8 ± 0* | 8 ± 1* | 8 ± 1** | 8 ± 1*** | 8 ± 1** |  |  |  |
| Instrumental V_D_ (n=6) | 9 ± 1 | 9 ± 1 | 10 ± 1 | 10 ± 1 | 10 ± 1 |  |  |  |
| **Driving Pressure (cmH_2_O)** |  | | | | | | | |
| Injury (n=6) | 10 ± 2 | 11 ± 2 | 17 ± 5 | 14 ± 3 | 19 ± 4 | **<0.001** | **<0.001** | **<0.001** |
| FiCO2 (n=7) | 8 ± 1 | 9 ± 3 | 10 ± 2 | 9 ± 1 | 9 ± 1 |  |  |  |
| Low V_T_ (n=6) | 7 ± 1 | 8 ± 1 | 8 ± 1 | 9 ± 2 | 9 ± 3 |  |  |  |
| Instrumental V_D_ (n=6) | 11 ± 2 | 10 ± 2 | 12 ± 3 | 13 ± 3 | 14 ± 3 |  |  |  |
| **Respiratory System Compliance (ml/cmH_2_O)** |  | | | | | | | |
| Injury (n=6) | 42 ± 6 | 37 ± 6 | 26 ± 10 | 29 ± 9 | 22 ± 4 | **<0.001** | **0.004** | **<0.001** |
| FiCO2 (n=7) | 43 ± 7 | 36 ± 7 | 34 ± 4 | 35 ± 5 | 38 ± 6*** |  |  |  |
| Low V_T_ (n=6) | 33 ± 5* | 29 ± 4* | 27 ± 3 | 26 ± 3 | 24 ± 3 |  |  |  |
| Instrumental V_D_ (n=6) | 34 ± 5 | 36 ± 8 | 31 ± 7 | 28 ± 5 | 26 ± 6 |  |  |  |
| **Lung Compliance (ml/cmH_2_O)** |  | | | | | | | |
| Injury (n=6) | 73 ± 26 | 61 ± 24 | 39 ± 26 | 42 ± 17 | 29 ± 8 | **<0.001** | **0.006** | 0.271 |
| FiCO2 (n=7) | 103 ± 28 | 91 ± 28 | 78 ± 29 | 67 ± 20 | 79 ± 26 |  |  |  |
| Low V_T_ (n=6) | 58 ± 9 | 53 ± 18 | 49 ± 14 | 45 ± 14 | 37 ± 9 |  |  |  |
| Instrumental V_D_ (n=6) | 61 ± 18 | 92 ± 70 | 60 ± 30 | 58 ± 30 | 44 ± 16 |  |  |  |
| **Chest Wall Compliance (ml/cmH_2_O)** |  | | | | | | | |
| Injury (n=6) | 123 ± 49 | 138 ± 61 | 126 ± 56 | 113 ± 39 | 94 ± 27 | 0.240 | **0.017** | **0.041** |
| FiCO2 (n=7) | 79 ± 17 | 64 ± 12 | 65 ± 6 | 78 ± 12 | 80 ± 14 |  |  |  |
| Low V_T_ (n=6) | 88 ± 34 | 81 ± 29 | 76 ± 29 | 73 ± 28 | 85 ± 35 |  |  |  |
| Instrumental V_D_ (n=6) | 82 ± 15 | 79 ± 31 | 85 ± 28 | 66 ± 26 | 70 ± 11 |  |  |  |
| **Gas Exchange** | | | | | | | | |
| **PaO_2_/FiO_2_** |  | | | | | | | |
| Injury (n=6) | 511 ± 56 | 504 ± 43 | 334 ± 163 | 450 ± 74 | 320 ± 128 | **<0.001** | **<0.001** | **<0.001** |
| FiCO2 (n=7) | 471 ± 52 | 450 ± 55 | 485 ± 68 | 495 ± 41 | 478 ± 96 |  |  |  |
| Low V_T_ (n=6) | 354 ± 65** | 335 ± 88** | 335 ± 61 | 332 ± 115 | 347 ± 66 |  |  |  |
| Instrumental V_D_ (n=6) | 419 ± 76 | 379 ± 61** | 288 ± 109 | 244 ± 111* | 269 ± 94 |  |  |  |
| **PaCO_2_ (mmHg)** |  | | | | | | | |
| Injury (n=6) | 39 ± 5 | 34 ± 3 | 39 ± 8 | 34 ± 5 | 37 ± 7 | 0.325 | **<0.001** | 0.082 |
| FiCO2 (n=7) | 70 ± 10*** | 65 ± 10*** | 64 ± 8*** | 63 ± 7*** | 63 ± 9*** |  |  |  |
| Low V_T_ (n=6) | 88 ± 25* | 93 ± 35* | 83 ± 21** | 85 ± 21** | 83 ± 20** |  |  |  |
| Instrumental V_D_ (n=6) | 83 ± 10*** | 90 ± 10*** | 81 ± 11*** | 95 ± 17*** | 85 ± 17** |  |  |  |
| **pH** |  | | | | | | | |
| Injury (n=6) | 7.47 ± 0.03 | 7.50 ± 0.02 | 7.45 ± 0.09 | 7.50 ± 0.04 | 7.45 ± 0.06 | **<0.001** | **<0.001** | **<0.001** |
| FiCO2 (n=7) | 7.28 ± 0.06*** | 7.34 ± 0.03*** | 7.39 ± 0.03 | 7.43 ± 0.03* | 7.41 ± 0.06 |  |  |  |
| Low V_T_ (n=6) | 7.20 ± 0.08** | 7.23 ± 0.13** | 7.31 ± 0.07* | 7.33 ± 0.08** | 7.37 ± 0.07 |  |  |  |
| Instrumental V_D_ (n=6) | 7.22 ± 0.05*** | 7.24 ± 0.04*** | 7.33 ± 0.06* | 7.30 ± 0.08** | 7.35 ± 0.06* |  |  |  |
| **HCO_3_^-^** |  | | | | | | | |
| Injury (n=6) | 28 ± 2 | 26 ± 2 | 26 ± 2 | 26 ± 2 | 25 ± 3 | **<0.001** | **<0.001** | **<0.001** |
| FiCO2 (n=7) | 31 ± 3 | 33 ± 4** | 37 ± 4*** | 40 ± 1*** | 38 ± 2*** |  |  |  |
| Low V_T_ (n=6) | 32 ± 3 | 35 ± 2*** | 39 ± 4*** | 43 ± 3*** | 47 ± 3*** |  |  |  |
| Instrumental V_D_ (n=6) | 31 ± 3 | 37 ± 1*** | 41 ± 2*** | 45 ± 1*** | 45 ± 3*** |  |  |  |
| **EtCO_2_ (mmHg)** |  | | | | | | | |
| Injury (n=6) | 34 [30 – 38] | 32 [30 – 35] | 31 [28 – 35] | 29 [27 – 36] | 29 [24 – 31] | **0.043** | **<0.001** | 0.359 |
| FiCO2 (n=7) | 69 [66 – 74]*** | 69 [65 – 77]*** | 68 [53 – 70]*** | 66 [63 – 71]*** | 67 [63 – 71]*** |  |  |  |
| Low V_T_ (n=6) | 73 [64 – 94]** | 84 [59 – 118]* | 82 [66 – 97]** | 67 [66 – 101]** | 80 [65 – 98]* |  |  |  |
| Instrumental V_D_ (n=6) | 76 [72 – 88]*** | 92 [84 – 102]*** | 81 [66 – 91]** | 90 [77-98]*** | 80 [70 – 93]*** |  |  |  |
| **Hemodynamics** | | | | | | | | |
| **Systolic Arterial Pressure (mmHg)** |  | | | | | | | |
| Injury (n=6) | 138 ± 4 | 130 ± 7 | 115 ± 9 | 114 ± 13 | 118 ± 7 | **<0.001** | **0.024** | 0.382 |
| FiCO2 (n=7) | 126 ± 11 | 123 ± 8 | 108 ± 3 | 110 ± 8 | 107 ± 10 |  |  |  |
| Low V_T_ (n=6) | 133 ± 12 | 130 ± 9 | 112 ± 13 | 105 ± 8 | 110 ± 8 |  |  |  |
| Instrumental V_D_ (n=6) | 131 ± 8 | 127 ± 7 | 123 ± 10 | 117 ± 12 | 119 ± 12 |  |  |  |
| **Diastolic Arterial Pressure (mmHg)** |  | | | | | | | |
| Injury (n=6) | 103 ± 8 | 94 ± 11 | 76 ± 11 | 77 ± 17 | 81 ± 9 | **<0.001** | **0.041** | 0.531 |
| FiCO2 (n=7) | 87 ± 11* | 79 ± 10 | 66 ± 7 | 66 ± 9 | 63 ± 13* |  |  |  |
| Low V_T_ (n=6) | 97 ± 14 | 83 ± 17 | 68 ± 31 | 57 ± 12 | 63 ± 12* |  |  |  |
| Instrumental V_D_ (n=6) | 93 ± 6 | 84 ± 10 | 79 ± 10 | 65 ± 14 | 63 ± 13 |  |  |  |
| **Mean Arterial Pressure (mmHg)** |  | | | | | | | |
| Injury (n=6) | 121 ± 5 | 113 ± 9 | 95 ± 9 | 94 ± 16 | 100 ± 8 | **<0.001** | 0.106 | 0.124 |
| FiCO2 (n=7) | 105 ± 12* | 100 ± 10 | 87 ± 4 | 88 ± 9 | 86 ± 13* |  |  |  |
| Low V_T_ (n=6) | 116 ± 13 | 96 ± 31 | 85 ± 14 | 80 ± 9 | 86 ± 10* |  |  |  |
| Instrumental V_D_ (n=6) | 113 ± 7 | 105 ± 10 | 101 ± 10 | 85 ± 13 | 78 ± 26 |  |  |  |
| **Systolic Pulmonary Artery Pressure (mmHg)** |  | | | | | | | |
| Injury (n=6) | 28 ± 5 | 29 ± 5 | 37 ± 7 | 30 ± 6 | 39 ± 6 | 0.419 | 0.875 | **<0.001** |
| FiCO2 (n=7) | 32 ± 3 | 33 ± 3 | 31 ± 2 | 30 ± 4 | 31 ± 4 |  |  |  |
| Low V_T_ (n=6) | 36 ± 6 | 34 ± 8 | 31 ± 6 | 31 ± 8 | 31 ± 4 |  |  |  |
| Instrumental V_D_ (n=6) | 31 ± 4 | 33 ± 4 | 31 ± 5 | 36 ± 2 | 34 ± 4 |  |  |  |
| **Diastolic Pulmonary Artery Pressure (mmHg)** |  | | | | | | | |
| Injury (n=6) | 15 ± 3 | 14 ± 3 | 18 ± 4 | 14 ± 3 | 20 ± 7 | 0.669 | 0.926 | **0.002** |
| FiCO2 (n=7) | 17 ± 4 | 18 ± 3 | 16 ± 2 | 17 ± 3 | 15 ± 4 |  |  |  |
| Low V_T_ (n=6) | 20 ± 3* | 19 ± 5 | 16 ± 3 | 17 ± 3 | 16 ± 3 |  |  |  |
| Instrumental V_D_ (n=6) | 15 ± 4 | 16 ± 7 | 15 ± 5 | 18 ± 5 | 18 ± 5 |  |  |  |
| **Mean Pulmonary Artery Pressure (mmHg)** |  | | | | | | | |
| Injury (n=6) | 22 ± 3 | 22 ± 4 | 27 ± 6 | 22 ± 4 | 30 ± 6 | 0.501 | 0.888 | **<0.001** |
| FiCO2 (n=7) | 25 ± 3 | 25 ± 2 | 24 ± 2 | 23 ± 3 | 23 ± 3 |  |  |  |
| Low V_T_ (n=6) | 28 ± 4* | 27 ± 5 | 23 ± 3 | 23 ± 4 | 24 ± 3 |  |  |  |
| Instrumental V_D_ (n=6) | 23 ± 4 | 25 ± 5 | 24 ± 5 | 27 ± 2 | 25 ± 5 |  |  |  |
| **Wedge Pressure (mmHg)** |  | | | | | | | |
| Injury (n=6) | 9 ± 3 | 8 ± 3 | 11 ± 3 | 9 ± 2 | 11 ± 3 | 0.416 | 0.085 | 0.226 |
| FiCO2 (n=7) | 8 ± 3 | 8 ± 3 | 9 ± 2 | 9 ± 2 | 8 ± 1 |  |  |  |
| Low V_T_ (n=6) | 9 ± 3 | 8 ± 5 | 7 ± 4 | 8 ± 2 | 9 ± 2 |  |  |  |
| Instrumental V_D_ (n=6) | 5 ± 1 | 6 ± 2 | 6 ± 1* | 7 ± 2 | 8 ± 3 |  |  |  |
| **Cardiac Output (L/min)** |  | | | | | | | |
| Injury (n=6) | 4.6 [4.2 – 6.3] | 3.5 [3.2 – 4.7] | 4.3 [3.9 – 5.3] | 4.2 [3.8 – 5.3] | 4.4 [3.7 – 5.2] | **<0.001** | 0.189 | **0.017** |
| FiCO2 (n=7) | 4.0 [3.5 – 4.2]* | 4.1 [3.3 – 4.9] | 4.2 [3.8 – 4.6] | 3.9 [3.4 – 4.3] | 4.8 [4.4 – 6.0] |  |  |  |
| Low V_T_ (n=6) | 3.7 [3.2 – 6.1] | 4.4 [4.2 – 4.8] | 4.7 [4.4 – 5.4] | 4.7 [4.7 – 5.2] | 6.1 [5.3 – 6.1]* |  |  |  |
| Instrumental V_D_ (n=6) | 3.7 [4.1 – 3.4]* | 4.5 [3.8 – 5.0] | 5.1 [5.3 – 3.6] | 5.5 [6.1 – 4.7] | 6.2 [4.3 – 6.2] |  |  |  |
| **Pulmonary Vascular Resistance (dyne/s/cm^-5^)** |  | | | | | | | |
| Injury (n=6) | 213 ± 38 | 296 ± 64 | 294 ± 87 | 243 ± 63 | 343 ± 73 | **0.020** | 0.337 | **0.003** |
| FiCO2 (n=7) | 349 ± 35*** | 332 ± 79 | 278 ± 39 | 283 ± 55 | 248 ± 27 |  |  |  |
| Low V_T_ (n=6) | 366 ± 143 | 293 ± 125 | 232 ± 44 | 237 ± 85 | 233 ± 38* |  |  |  |
| Instrumental V_D_ (n=6) | 384 ± 86** | 343 ± 148 | 326 ± 83 | 303 ± 74 | 277 ± 64 |  |  |  |
| **Heart Rate (bpm)** |  | | | | | | | |
| Injury (n=6) | 108 ± 17 | 106 ± 20 | 108 ± 28 | 100 ± 27 | 98 ± 15 | 0.130 | 0.404 | **0.029** |
| FiCO2 (n=7) | 94 ± 5 | 97 ± 12 | 91 ± 12 | 92 ± 11 | 95 ± 21 |  |  |  |
| Low V_T_ (n=6) | 93 ± 23 | 121 ± 31 | 112 ± 23 | 105 ± 21 | 109 ± 23 |  |  |  |
| Instrumental V_D_ (n=6) | 85 ± 9 | 102 ± 12 | 99 ± 18 | 117 ± 21 | 120 ± 44 |  |  |  |
| **EIT data** | | | | | | | | |
| **V_TRIGHT/LEFT_** |  | | | | | | | |
| Injury (n=6) | 3.3 ± 0.72 | 4.7 ± 2.6 | 2.6 ± 1.5 | 2.7 ± 1.0 | 1.6 ± 0.6 | **0.047** | 0.197 | **0.005** |
| FiCO2 (n=7) | 1.8 ± 1.1 | 1.6 ± 1.2 | 1.6 ± 0.5 | 1.7 ± 0.3 | 1.8 ± 0.4 |  |  |  |
| Low V_T_ (n=6) | 2.6 ± 1.7 | 2.3 ± 1.2 | 2.7 ± 1.7 | 3.3 ± 2.3 | 2.5 ± 1.0 |  |  |  |
| Instrumental V_D_ (n=6) | 3.5 ± 2.2 | 2.6 ± 1.0 | 2.2 ± 1.1 | 2.1 ± 0.8 | 2.2 ± 0.9 |  |  |  |
| **Perfusion_LEFT_ %** |  | | | | | | | |
| Injury (n=6) | 16 ± 4 | 17 ± 3 | 18 ± 5 | 18 ± 3 | 16 ± 4 | 0.388 | **<0.001** | 0.968 |
| FiCO2 (n=7) | 31 ± 7** | 33 ± 11* | 33 ± 10* | 27 ± 5* | 28 ± 12 |  |  |  |
| Low V_T_ (n=6) | 25 ± 5* | 22 ± 9 | 21 ± 8 | 20 ± 3 | 21 ± 8 |  |  |  |
| Instrumental V_D_ (n=6) | 23 ± 4 | 21 ± 3 | 20 ± 4 | 19 ± 4 | 18 ± 4 |  |  |  |
| **Compliance RS_RIGHT_ (ml/cmH_2_O)** |  | | | | | | | |
| Injury (n=6) | 33 ± 5 | 32 ± 2 | 23 ± 11 | 25 ± 2 | 14 ± 4 | **<0.001** | 0.181 | **<0.001** |
| FiCO2 (n=7) | 27 ± 7 | 21 ± 5** | 20 ± 5 | 22 ± 4 | 25 ± 5* |  |  |  |
| Low V_T_ (n=6) | 22 ± 3* | 19 ± 5** | 19 ± 4 | 18 ± 5 | 17 ± 3 |  |  |  |
| Instrumental V_D_ (n=6) | 25 ± 7 | 26 ± 6 | 21 ± 6 | 19 ± 5 | 18 ± 6 |  |  |  |
| **Compliance RS_LEFT_ (ml/cmH_2_O)** |  | | | | | | | |
| Injury (n=6) | 10 ± 2 | 8 ± 4 | 9 ± 2 | 10 ± 2 | 9 ± 2 | 0.151 | **0.004** | 0.693 |
| FiCO2 (n=7) | 17 ± 6 | 17 ± 6 | 14 ± 2 | 13 ± 1 | 14 ± 3* |  |  |  |
| Low V_T_ (n=6) | 11 ± 4 | 10 ± 3 | 9 ± 4 | 7 ± 3 | 7 ± 2 |  |  |  |
| Instrumental V_D_ (n=6) | 9 ± 4 | 11 ± 4 | 10 ± 3 | 9 ± 3 | 9 ± 3 |  |  |  |

Data are expressed as mean ± SD or median [quartiles], as appropriate. Statistical analysis was performed using two-way ANOVA for repeated measurements or mixed effects analysis, including group and time as main independent factors and group-by-time interaction.

*p<0.05, **p<0.01 and ***p<0.001 vs Injury group.

Abbreviations: PaO_2_/FiO_2_: partial pressure of oxygen / inspired fraction of oxygen; PaCO_2_: partial pressure of carbon dioxide; EtCO_2_: end-tidal CO_2_; V_TRIGHT/LEFT_: ratio between tidal volume distending the right and the left lung; Perfusion_LEFT_: percentage of perfusion to the left lung; Compliance RS_RIGHT_: right-side regional compliance of the respiratory system; Compliance RS_LEFT_: left-side regional compliance of the respiratory system right lung.

**Figure S1**


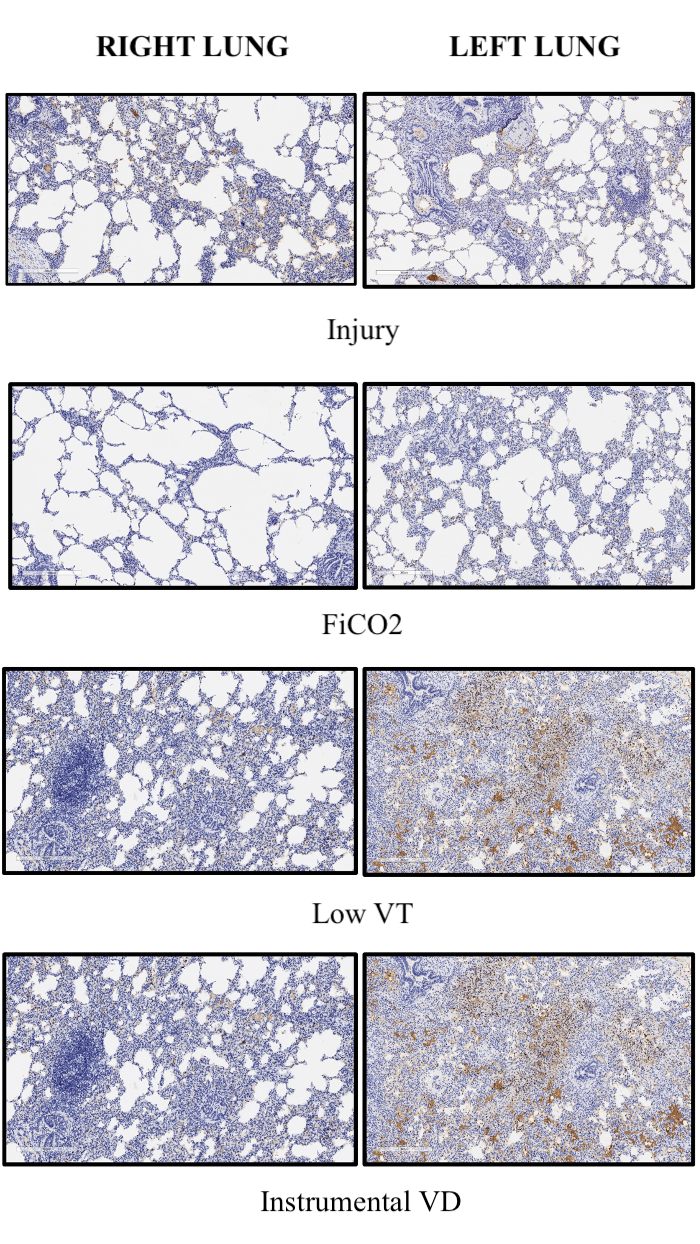


**Figure S2**


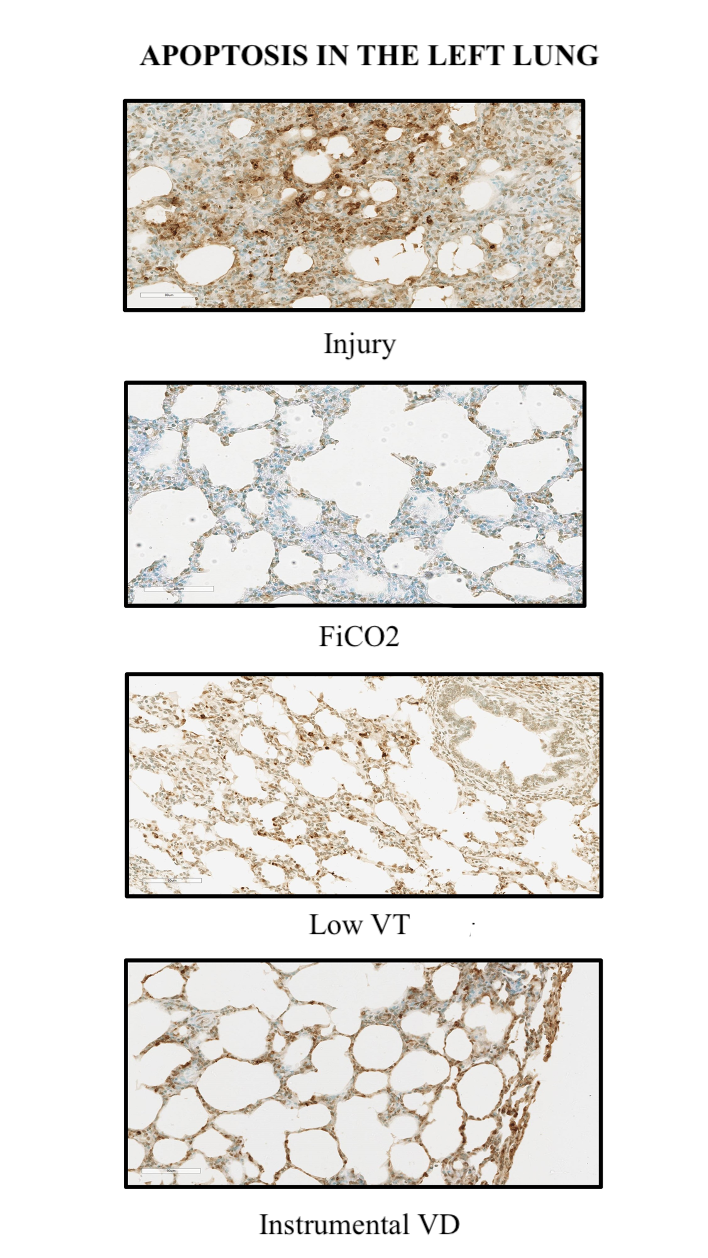

Supplement: Supplementary file 1 [file Data_Sheet_1.docx]
